# Exposure to a Highly Caloric Palatable Diet During Pregestational and Gestational Periods Affects Hypothalamic and Hippocampal Endocannabinoid Levels at Birth and Induces Adiposity and Anxiety-Like Behaviors in Male Rat Offspring

**DOI:** 10.3389/fnbeh.2015.00339

**Published:** 2016-01-06

**Authors:** María Teresa Ramírez-López, Mariam Vázquez, Laura Bindila, Ermelinda Lomazzo, Clementine Hofmann, Rosario Noemí Blanco, Francisco Alén, María Antón, Juan Decara, Daniel Ouro, Laura Orio, Juan Suarez, Beat Lutz, Fernando Rodríguez de Fonseca, Raquel Gómez de Heras

**Affiliations:** ^1^Departamento de Psicobiología, Facultad de Psicología, Universidad Complutense de MadridMadrid, Spain; ^2^Unidad de Gestión Clínica de Salud Mental, Instituto IBIMA, Hospital Regional Universitario de Málaga, Universidad de MálagaMálaga, Spain; ^3^Institute of Physiological Chemistry, University Medical Center of the Johannes Gutenberg University of MainzMainz, Germany

**Keywords:** maternal diet, endocannabinoids, adipogenesis, anxiety, development, hypothalamus, hippocampus

## Abstract

Exposure to unbalanced diets during pre-gestational and gestational periods may result in long-term alterations in metabolism and behavior. The contribution of the endocannabinoid system to these long-term adaptive responses is unknown. In the present study, we investigated the impact of female rat exposure to a hypercaloric-hypoproteic palatable diet during pre-gestational, gestational and lactational periods on the development of male offspring. In addition, the hypothalamic and hippocampal endocannabinoid contents at birth and the behavioral performance in adulthood were investigated. Exposure to a palatable diet resulted in low weight offspring who exhibited low hypothalamic contents of arachidonic acid and the two major endocannabinoids (anandamide and 2-arachidonoylglycerol) at birth. Palmitoylethanolamide, but not oleoylethanolamide, also decreased. Additionally, pups from palatable diet-fed dams displayed lower levels of anandamide and palmitoylethanolamide in the hippocampus. The low-weight male offspring, born from palatable diet exposed mothers, gained less weight during lactation and although they recovered weight during the post-weaning period, they developed abdominal adiposity in adulthood. These animals exhibited anxiety-like behavior in the elevated plus-maze and open field test and a low preference for a chocolate diet in a food preference test, indicating that maternal exposure to a hypercaloric diet induces long-term behavioral alterations in male offspring. These results suggest that maternal diet alterations in the function of the endogenous cannabinoid system can mediate the observed phenotype of the offspring, since both hypothalamic and hippocampal endocannabinoids regulate feeding, metabolic adaptions to caloric diets, learning, memory, and emotions.

## Introduction

Obesity and overweight prevalence are increasing worldwide in adults and children. Specifically, 11% of men and 15% of women were obese in 2014, whereas 6.7% of children were overweight in 2013 (World Health Organization, 2014). Additionally, obesity could be associated to the metabolic syndrome, which is defined as a combination of several risk factors that include hyperglycemia, increased blood pressure, elevated triglyceride levels, and low high-density lipoprotein cholesterol levels, besides central obesity (Alberti et al., 2009). This syndrome increases the risk of suffering from diabetes mellitus and cardiovascular diseases (Kaur, 2014). Furthermore, obesity has been linked to psychiatric disorders such as depression or anxiety, particularly when obese subjects have an unhealthy metabolic profile (Stunkard et al., 2003; Phillips and Perry, 2015).

Although lifestyle factors, such as highly palatable and nutrient-poor junk foods and reduced physical activity, play an important role in the etiology of obesity and metabolic syndrome, several epidemiological and experimental studies have pointed out that maternal malnutrition during critical periods of life could increase the vulnerability in offspring of suffering from metabolic diseases (Hales and Barker, 1992). This vulnerability can be extended to the incidence of behavioral disorders (Sullivan et al., 2014) later in life. The term which describes this process is known as nutritional programming (Lucas, 1991). In this regard and considering that maternal and postnatal nutrition tend to be excessive in Western societies, it has been shown that maternal obesity is commonly associated with macrosomy at birth (Baeten et al., 2001; Boney et al., 2005; Bhattacharya et al., 2007) and high risk of suffering from metabolic syndrome in adulthood (Danielzik et al., 2002; Boney et al., 2005; Catalano et al., 2009; Pirkola et al., 2010). Additionally, animal models have also shown that exposure to a hypercaloric diet or junk food (also known as cafeteria diet) during the perinatal period could alter various metabolic parameters later in life (Khan et al., 2005; Taylor et al., 2005; Srinivasan et al., 2006; Bayol et al., 2007; Samuelsson et al., 2008; Shankar et al., 2008; Howie et al., 2009; Kirk et al., 2009; White et al., 2009; Ong and Muhlhausler, 2011; Dahlhoff et al., 2014), even when mothers do not necessarily become obese (Guo and Jen, 1995; Langley-Evans, 1996; Comstock et al., 2012).

Furthermore, the exposure to hypercaloric diets and/or junk food have been associated to behavioral abnormalities, such as hyperphagia (Bayol et al., 2007; Samuelsson et al., 2008; Howie et al., 2009; Kirk et al., 2009; Ong and Muhlhausler, 2011), high preference for palatable food (Bayol et al., 2007; Ong and Muhlhausler, 2011) or food neophobia (Peleg-Raibstein et al., 2012). In addition, emotional behavior has been shown to be altered by a maternal hypercaloric diet or cafeteria diet (Sullivan et al., 2010; Wright et al., 2011; Peleg-Raibstein et al., 2012; Sasaki et al., 2013, 2014).

Several mechanisms associated with the process of malprogramming, which occurs during the perinatal period (Srinivasan et al., 2006), have been identified, such as oxidative stress after the exposure to hypercaloric or cafeteria diets (Bouanane et al., 2009), epigenetic modifications (Vucetic et al., 2011), and alterations in the hypothalamic-pituitary adrenal (HPA) axis (Sasaki et al., 2013, 2014). Additionally, modifications in hypothalamic neuronal circuitries have been shown. Indeed, maternal high fat diet increases the number of neurons expressing orexigenic peptides and elevates the levels of mRNA of these neuropeptides in the offspring (Muhlhausler et al., 2006; Chang et al., 2008; Chen et al., 2009; Gupta et al., 2009; Stachowiak et al., 2013). Considering that the hypothalamus plays an important role in energy homeostasis, appetite, and body development, these findings may explain the long-term changes associated to eating habits and adiposity distribution. Furthermore, leptin, an important hormone secreted by white adipocytes, has been linked to the development of hypothalamus (Bouret et al., 2004; Cottrell et al., 2009). The dysregulation of this adipose tissue hormone has been documented in several studies, suggesting that leptin is critical for the establishment of developmental programming (Vickers and Sloboda, 2012). Additionally, alterations in opioid and dopaminergic signaling in the central reward pathway after the exposure to junk food have been described (Ong and Muhlhausler, 2011).

To date, the role of the endocannabinoid system in the process of nutritional programming has not yet been understood, even though it is tightly connected to other mechanisms that have been widely studied such as leptin (Di Marzo et al., 2001), opioid and dopaminergic systems (Cota et al., 2006, 2007). Endocannabinoids are signaling lipids produced from membrane long-chain fatty acids in response to neuronal activity (Mackie, 2008). The endocannabinoid system is particularly important in the regulation of energy metabolism, feeding behavior and energy homeostasis (Alen et al., 2013; Cristino et al., 2014). The dysregulation of this system has been associated to the development of obesity (Matias and Di Marzo, 2007; Bermudez-Silva et al., 2010) and neuropsychiatric diseases as well (Lutz, 2009).

The two main endocannabinoids, N-arachidonoylethanolamide (anandamide, AEA) and 2-arachidonoylglycerol (2-AG), are produced from arachidonic acid (AA) (Hansen and Artmann, 2008). Several studies have shown that altered nutritional conditions can modify endocannabinoid levels (Kirkham et al., 2002; Hanus et al., 2003; Alvheim et al., 2014), but unfortunately, only a few studies have investigated the effects of nutritional changes during the perinatal period. For instance, it has been shown that milk deficient of AA during lactation as well as maternal undernutrition decrease the levels of endocannabinoids in the brain during lactation or at weaning (Berger et al., 2001; Matias et al., 2003). Additionally, it has been found that the composition in fatty acids of the maternal diet produces changes in the endocannabinoids levels in the neonatal hypothalamus and hippocampus (D'Asti et al., 2010). Furthermore, it has been shown that lifelong n-3 polyunsaturated fatty acids (PUFAs) dietary insufficiency, from the perinatal period to adulthood, is associated to impairment in endocannabinoid-mediated neuronal functions in the adult brain (Lafourcade et al., 2011), and these modifications led to behavioral abnormalities (Lafourcade et al., 2011; Larrieu et al., 2012). In line with these findings, it has been recently proposed that maternal nutrition can alter endocannabinoid signaling and, thus, this system could act as a molecular substrate of developmental programming (Keimpema et al., 2013). However, further investigations are necessary to clarify the role of the endocannabinoid system in this process.

In the present study, we investigated the effects of the exposure of female rats to a cafeteria diet (junk food) on neonatal outcomes and the levels of endocannabinoids and N-acylethanolamides in the hypothalamus of offspring at birth. Furthermore, offspring were monitored until adulthood, and the metabolic indices and behavioral parameters were examined. We propose that altered endocannabinoid levels at birth may reflect intrauterine changes due to the type of maternal diet, which may ultimately induce a metabolic imbalance in the offspring. Accordingly, we hypothesize that a maternal highly palatable diet may increase the risk in the offspring of suffering from metabolic diseases or psychiatric disorders later in life.

## Materials and methods

### Animals, diets, and experimental design

This study was approved by the Animal Ethics Committee of the Complutense University of Madrid and was conducted in compliance with the European Directive 2010/63/EU on the protection of animals used for scientific purposes and in accordance with the current Spanish regulations (RD 53/2013 and 178/2004).

Experiments were performed initially in prepuberal female Wistar rats (Harlan, Barcelona, Spain). Animals were allowed to acclimate for 3 weeks before diet assignation. Rats were handled and individually housed at a 12 h light-dark cycle with temperature of 22 ± 1°C. After the acclimatization period, animals were weighed (average weight: 179 ± 4 g) and randomly assigned to control (*n* = 9) or free-choice diet (*n* = 11) animals. At this stage, there was not a statistically significant difference in body weight among groups.

Control rats were given free access to standard chow (standard chow SAFE A04, Panlab, Barcelona, Spain) and water. In contrast, free-choice rats were allowed to choose between standard rat chow (standard chow SAFE A04, Panlab, Barcelona, Spain) and a highly palatable food composed of a mixture of chocolates (cafeteria diet). Both types of food and water were provided *ad libitum* in both animal groups. The mixture of chocolate food was composed of a homogenous mixture of Mars®, Snickers®, Bounty®, and Milka® in equal proportions as previously described (Heyne et al., 2009; Martin-Garcia et al., 2010). A detailed nutritional description of the composition of both types of food is shown in Table 1.

**Table 1:** **Composition of the diets used in the present study**.

	**Standard chow diet**	**Highly palatable diet**
Protein %	16.1%	6%
Carbohydrate %:	60%	60.4%
• Simple carbohydrates	3.3%	89%
Fat %:	3.1%	24.45%
• Saturated fatty acids	22.20%	56.2%
• Unsaturated fatty acids	77.70%	43.88%
Fiber %	4%	1.45%
Sodium %	0.0025%	0.17%
Energy	2.9 Kcal/g	4.88 Kcal/g

During the pregestational period, food intake and weight were measured weekly. The estrous cycle was evaluated daily starting from 2 weeks before mating to check cycle regularity. Eight weeks after the beginning of diet assignation, females were allowed to mate with males of the same strain in their home cage for 24 h at the beginning of the proestrous cycle. Each male rat was mated with females from both control and free-choice groups. The presence of a vaginal plug or spermatozoa in the vaginal smear the following morning confirmed successful mating, and this was designated as gestational day 0. During the gestational period, food intake and weight were measured daily and female rats were maintained on the same diet paradigm as in the pregestational period.

The day in which the litter was born was defined as postnatal day 0 (PN0). Within 14 h after birth, pups were weighed and sexed. The vitality of the pups was observed in every litter as well as the presence of prominent “milkbands” in pup stomachs, which is an indicative sign of adequate milk ingestion (Fride et al., 2003). The number of litters which presented pups without prominent milkbands was recorded for every experimental group. Litter size was arranged to comprise up to 8 pups, consisting of 4 males and 4 females. The remaining pups were quickly sacrificed by decapitation and brains were collected for further endocannabinoid measurement.

Rat dams were exposed to their respective diets throughout the lactation period. Food intake and dams/pups weight were measured 3 days per week during this period. At PN day 22–23, offspring from both animal groups were weaned and exposed to standard chow diet (*n* = 15 and *n* = 17 for control and free-choice perinatal diet groups, respectively). Rats belonging to the same litter from each perinatal diet group were housed together (2–3 rats/cage) where possible. Dams were sacrificed.

**Figure 1:**
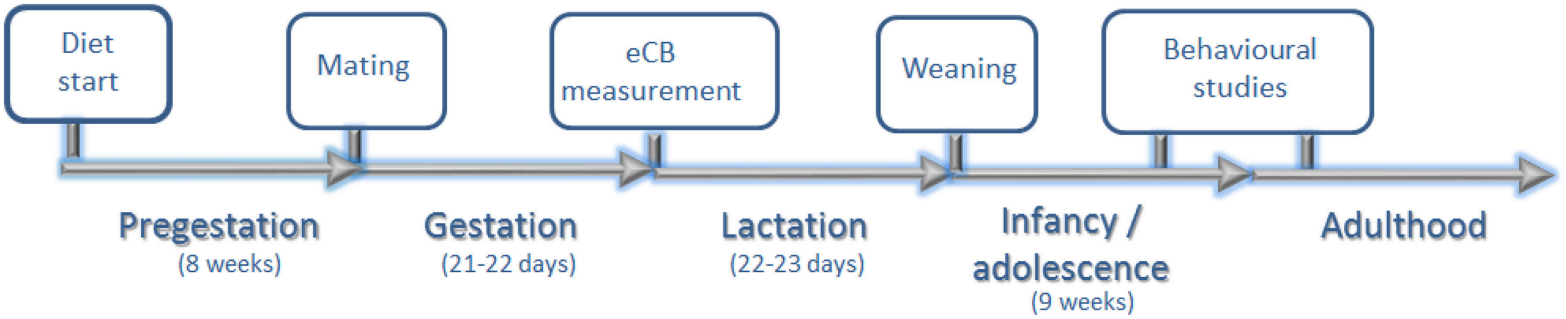
**Experimental design**. Eight weeks after diet assignation, rat dams were allowed to mate with males of the same strain. At PN day 0, endocannabinoid (eCB) levels extracted from the hypothalamus and hippocampus of male offspring were measured. Rat dams continued on the respectively assigned diet for the entire perinatal period (pre-gestation, gestation, and lactation). At PN days 22–23, all offspring were weaned and exposed to a standard chow diet. Behavioral studies (elevated plus maze, open field and chocolate preference test) were performed in adolescence (8th PN week). Chocolate preference was re-measured again in adulthood (12–13th PN weeks).

During the post-weaning period, weight and food intake were measured weekly. The total food intake from each cage was measured and equally divided according to the number of pups per cage to calculate individual food intake. Behavioral studies were performed in adolescence and adulthood (Figure 1). At the 5th postnatal month, three quarters of the male offspring were sacrificed.

All experiments were carried out exclusively for male offspring. The term “perinatal” was used here to refer to pregestational, gestational and lactation periods. We use the criteria described by Vickers to refer to different age stages (Vickers et al., 2000).

### Evaluation of caloric intake, nutritional intake, and adiposity

Food intake was determined by subtracting the amount of each food type left in the cage from the total amount of food provided. Comparisons among groups were carried out by calculating cumulative caloric intake (Kcal/Kg) as well as weight gain in each period of the study.

Nutritional intake was evaluated by calculating food intake of both types of food (g) and the percentage of nutrients of the specific food provided, considering the data of Table 1. Comparisons among groups were performed by calculating cumulative intake (g) of proteins, carbohydrates and fats at the end of gestational and lactation periods (gestational day 20 and lactation day 21 respectively).

Adiposity was estimated by calculating the percentage of abdominal fat weight over total body weight at the time the animals were sacrificed. Rats were weighed immediately before death and sacrificed by decapitation after administration of Equitesin® (3 mg/kg). Then, perirenal and perigonadal fat deposits were dissected and weighed. The sum of both types of fat was used to determine the percentage of abdominal fat.

### Endocannabinoids measurement

At PN day 0, the male offspring chosen to be sacrificed were decapitated during the second/third hour of the dark phase and brains were quickly removed and frozen at −80°C until brain region isolation. To avoid litter effects, brains from at least three litters per group were used for endocannabinoid measurement (control pups *n* = 7–5 and free-choice pups *n* = 13–11, for hypothalamus and hippocampus respectively). For the isolation of the brain regions selected, brains were thawed in cold Tris-HCl buffer (50 mM, pH = 7.40) and the entire hypothalamus and right hippocampus were immediately isolated and frozen at −80°C until lipid extraction. The overall isolation procedure was carried out in less than 7 min for all animals to avoid *ex vivo* production/degradation of endocannabinoids.

For lipid extraction, pre-cooled steel balls of 5 mm were added to pre-cooled tubes containing the tissue. A solution of deuterated endocannabinoids (AEA-d4, 2-AG-d5, AA-d8, MAEA, OEA-d2, PEA-d4, and 1-AG-d5, Cayman Chemicals, Ann Arbor, MI, USA) in acetonitrile was added to the tissue along with 300 μl of ice-cold 0.1 M formic acid and 300 μl of ethylacetate/hexane (9:1, v/v). Then, the samples were homogenized with a TissueLyser II (Qiagen, Hilden, Germany) for 60 s at 30 Hz. Subsequently, the samples were centrifuged for 10 min at 5000 g and 4°C. The organic phase was removed and evaporated under a gentle stream of nitrogen at 37°C. The aqueous phase was further used for protein content determination. The lipid extract was re-dissolved in 50 μl acetonitrile/water (1:1, v/v) and quantitative analysis of the endocannabinoid levels was carried out by liquid chromatography-multiple reaction monitoring (LC-MRM). The concentrations of internal standards, as well as the calibration curves, were set and tailored using test hypothalamus and hippocampal tissues. LC/MRM conditions for quantitative analysis of the endocannabinoids were set as previously reported (Wenzel et al., 2013) and endocannabinoid levels were normalized to the corresponding protein content of the tissues.

For protein quantification, the BCA method (bicinchoninic acid assay) was used and the measurements performed on a FLUOstar Galaxy (BMG Labtechnologies).

### Behavioral studies

#### Elevated plus maze

In adolescence, at the 7–8th PN weeks, anxiety-related responses in handled animals were evaluated with the elevated plus maze. The elevated plus-maze (Panlab, Barcelona, Spain) consisted of a cross-shaped platform made of black and gray plastic. The platform was elevated 65 cm from the floor and had two opposing open arms (50 × 10 cm) and two closed arms of the same size. The closed arms were fenced by 50-cm high opaque walls. A central area of 10 cm^2^ connected all arms. The light intensity was set up at 150 lux in the open arms and 80 lux in the closed arms. The test was performed after 5 h after the beginning of the dark phase. When the test started, each rat was placed in the central area facing one of the open arms and opposite to the experimenter position. Then, the animal was allowed to explore the maze freely for 5 min. After, the rat was moved back to the home cage and the maze was cleaned. The number of open arm and closed arm entries and the percentage of time spent on open and closed arms were determined by a computer-controlled system recording the interruptions of infrared photo beams located along each arm. Data were analyzed by using the MAZEsoft software (Panlab, Barcelona, Spain). Animals that fell off the maze during the test were excluded from the analysis.

#### Open field

In the 2 days following the elevated plus maze test, locomotor activity and anxiety related responses were evaluated with the open field test. The open field consisted of a square arena (80 cm × 80 cm and 40 cm high) virtually divided into a peripheral zone and a central zone (40 cm × 40 cm). It was made of plywood and was located in an experimental room illuminated with low light intensity (30 lux). The test was performed 5 h after the beginning of the dark phase. Each rat was positioned in the center of the open field and was allowed to explore freely for 5 min. After, the rat was moved back to the home cage and the arena was cleaned. A video camera installed above the arena was connected to a monitor and a video tracking motion analysis system (Smart, Panlab, Harvard Apparatus, Spain), which measured the total distance traveled (cm) and mean speed (cm/s). The program calculated the percentage of time spent on central area as well as the number of entries to the center zone as an index of anxiety-like behavior.

#### Chocolate preference test

The chocolate preference test was performed in adolescence (8–9th PN weeks) and was repeated again in adulthood (12–13th PN weeks). At the beginning of the test, animals were single-housed in new cages provided with both types of food (standard chow and the mixture of chocolates) and water *ad libitum*. Food intake for both types of food and animal weight was determined 24 h after the beginning of the test. Chocolate preference was calculated as the percentage of chocolate eaten over total food provided.

#### Statistical analysis

All data are expressed as mean ± SEM. Statistical analysis of results was performed by using the GraphPad Prism version 5.0 program (GraphPad Software Inc., San Diego, CA, USA) and SPSS15.0 for windows (SPSS Inc., Chicago, IL, USA). Weight gain over the time and caloric intake were analyzed by One-way repeated measures analysis of variance (ANOVA). Multiple comparisons were assessed by Bonferroni *post-hoc* test. To assess the differences among groups the presence of prominent milkbands, the chi-squared test was performed. Differences in endocannabinoid levels were analyzed by using the U Mann Whitney test. The Kaplan Meier survival analysis and log-rank test were adopted to analyze diet-induced differences in the survival rate among the experimental groups. Results from the chocolate preference test were analyzed by Two-way ANOVA with group (control vs. free-choice animals) and age period used as variables. Further analyses were performed by using the Student *t*-test, when data passed normality requirements (D'Agostino-Pearson test), or U Mann Whitney test. A *p*-value below 0.05 was considered statistically significant.

## Results

### Effect of diet on rat dams during perinatal period

#### Weight gain and adiposity

**Figure 2:**
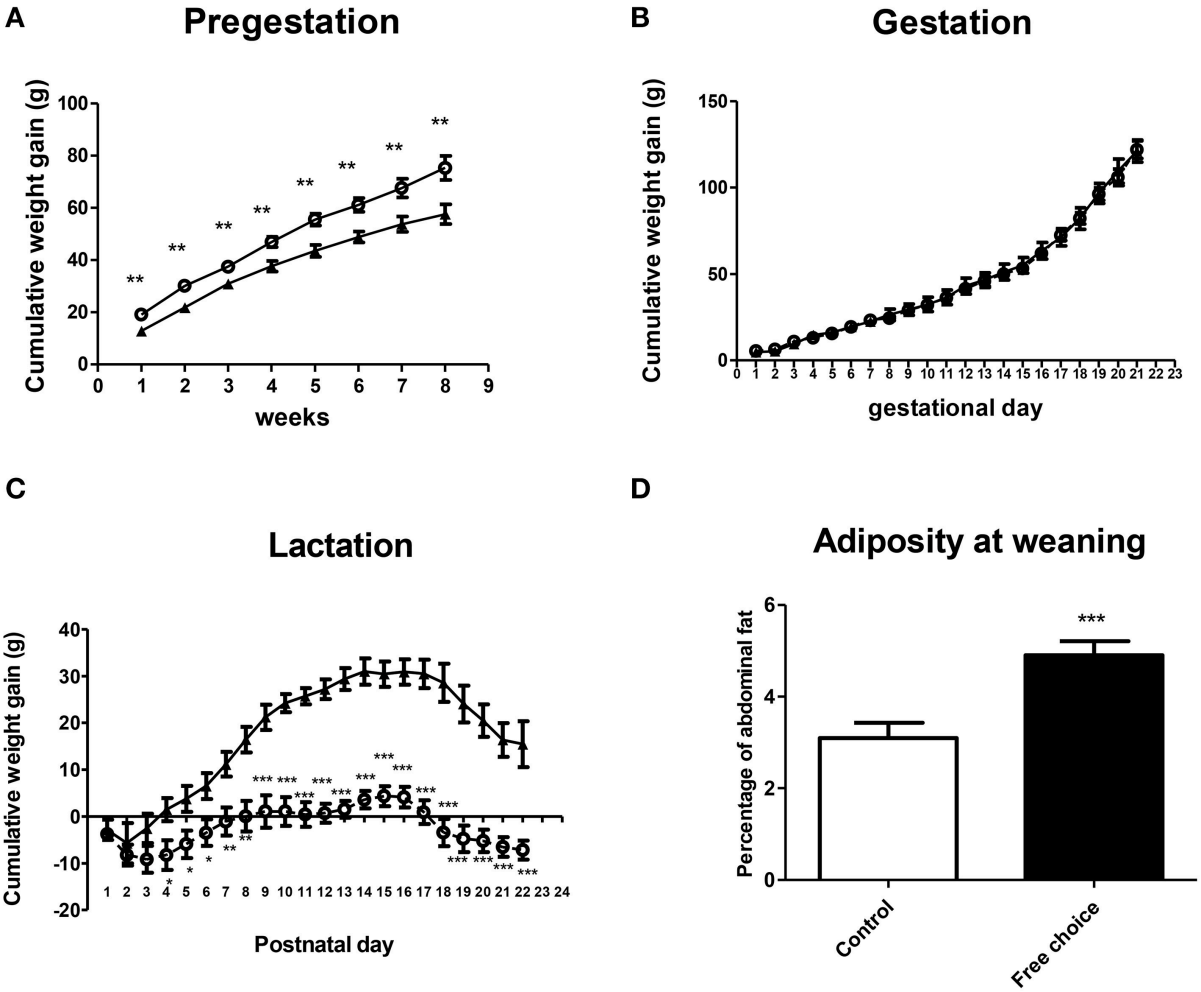
**Effect of diet on weight gain and adiposity of dams during perinatal period**. Values are expressed as mean ± SEM. ^*^p < 0.05, ^**^p < 0.01, ^***^p < 0.001. Cumulative weight gain (g) of control (solid triangles) and free-choice (open circles) dams during pregestation **(A)**, gestation **(B)**, and lactation **(C)**. Dams adiposity at weaning **(D)**: control (open bars) vs. free-choice (solid bars) dams.

Rat dams were exposed to their respective diets throughout the perinatal period (Figure 1). Repeated measures ANOVA revealed that dams from free-choice group gained more weight compared with controls before pregnancy [*F*_(1, 19)_ = 14.844, *p* = 0.001] (Figure 2A), although no significant differences between control and free-choice groups in body weight at mating were found (data not shown). During the gestational period, the difference in weight gain between groups was not significant (Figure 2B). Interestingly, after birth and during lactation period, free-choice dams gained significantly less weight than controls [repeated measures ANOVA, *F*_(1, 18)_ = 41.836, *p* < 0.001] (Figure 2C).

Regarding adiposity, palatable dams showed higher percentage of abdominal fat than controls at the end of the lactation period (weaning day) (*t* = 3.961, *p* < 0.001) (Figure 2D), although no significant differences in body weight were detected during this period (data not shown).

#### Caloric intake during perinatal period

**Figure 3:**
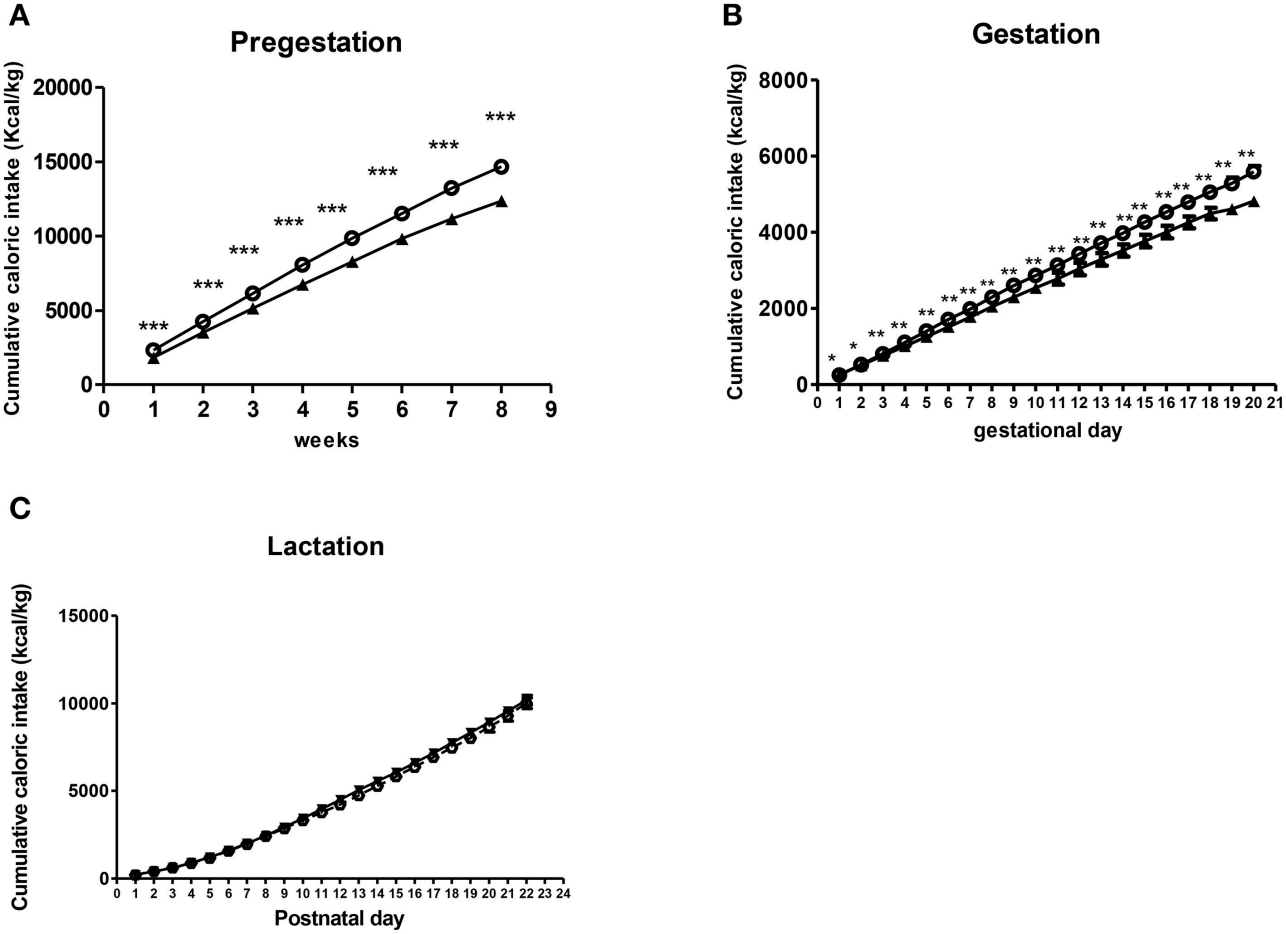
**Effect of diet on caloric intake of dams during perinatal period**. Values are expressed as mean ± SEM. ^*^p < 0.05, ^**^p < 0.01, ^***^p < 0.001. Cumulative caloric intake (kcal/kg) of control (solid triangles) and free-choice (open circles) dams during pregestation **(A)**, gestation **(B)**, and lactation **(C)**.

Repeated measures ANOVA showed that dams from the free-choice group had higher caloric intake than controls before pregnancy (pregestation) [*F*_(1, 19)_ = 28.607, *p* < 0.001] (Figure 3A), which lasted for the entire gestational period [*F*_(1, 17)_ = 15.099, *p* = 0.001] (Figure 3B). However, during the lactation period, no significant differences in caloric intake between groups were found (Figure 3C).

#### Nutritional intake during pregnancy and lactation

During pregnancy and lactation, free-choice dams consumed significantly less proteins (*t* = 12.61, *p* < 0.0001 and *t* = 10.93, *p* < 0.0001 respectively) and less carbohydrates (*t* = 3.983, *p* < 0.001 and *t* = 5.803, *p* < 0.0001, respectively) than controls (Supplementary Figure 1). In contrast, free-choice dams absorbed more fat than controls in gestation as well lactation period (*t* = 15.94, *p* < 0.0001 and *t* = 15.65, *p* < 0.0001, respectively) (Supplementary Figure 1).

### Effect of maternal diet on birth outcomes and mortality

Most of the pups from both groups were born between gestational days 21–22. Only in the free choice group, one litter was born at gestational day 20 and another litter at gestational day 23. Offspring from free-choice dams weighed significantly less than controls at birth either when analysis was done in both sexes together or in male and female separately (both sexes *t* = 9.552, *p* < 0.001, males *t* = 5.641, *p* < 0.001 and females *t* = 8.142, *p* < 0.001) (Supplementary Figure 2A). No significant differences in litter size for both sexes (control vs. free-choice) were found (Supplementary Figure 2B). The number of litters which presented some pups without prominent milkbands were significantly higher in free choice group, as revealed by the chi-squared test ($x^{2}df_{1}=5.454$, *p* < 0.05) (data not shown). Furthermore, a difference in the mortality rate between groups was found as 9.43% of male pups from the free-choice dams were dead at birth (4.31% of total newborn pups), whereas no casualties occurred in the control group (data not shown). Moreover, during the lactation period the Kaplan Meyer survival analysis and Log-rank test revealed higher mortality risk in offspring of both sexes from free-choice dams as compared to controls (Log-rank test: $x^{2}df_{1}=5.068$, *p* < 0.05) (Supplementary Figure 2C).

### Hypothalamic endocannabinoid and N-acylethanolamide levels of male offspring at birth

**Figure 4:**
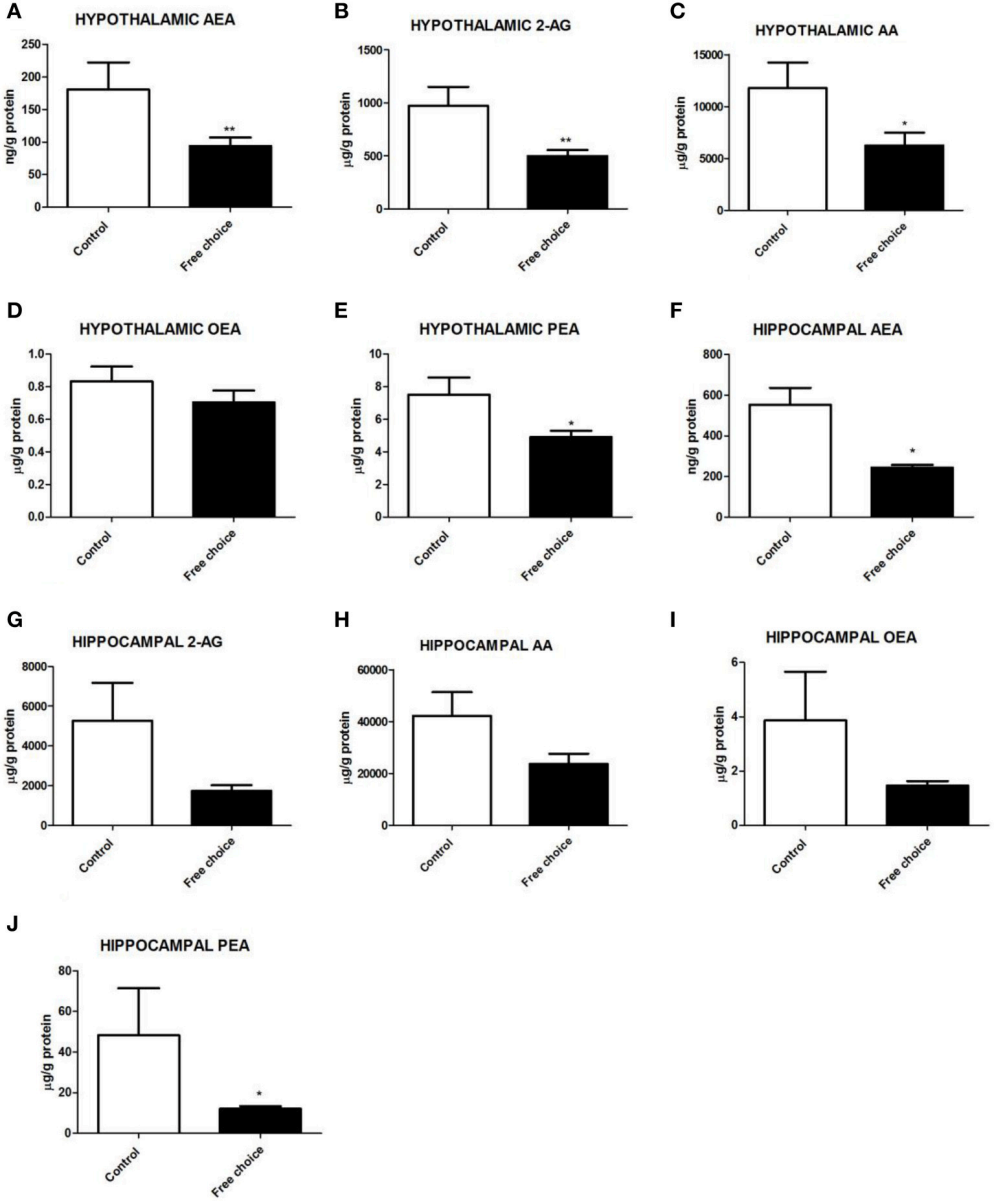
**Levels of endocannabinoids and N-acylethanolamides in the hypothalamus and hippocampus of male offspring**. Tissular contents (ng/g or mg/g of protein) of AEA **(A,F)**, 2-AG **(B,G)**, AA **(C,H)**, OEA **(D,I)**, and PEA **(E,J)** in the hypothalamus and hippocampus respectively of male pups born from mothers fed with control diet (open bars) or free-choice diet (solid bars). Values are expressed as mean ± SEM. ^*^p < 0.05, ^**^p < 0.01.

U Mann Whitney test analysis showed significant differences between the two groups in hypothalamic endocannabinoid levels at birth. Male pups from free-choice dams exhibited significantly lower levels of AEA (*U* = 9.000, *p* < 0.01), 2-AG (*U* = 10.00, *p* < 0.001), and AA (*U* = 13.00, *p* < 0.05) (Figures 4A–C, respectively). Palmitoylethanolamide (PEA) levels were also significantly decreased in offspring from free-choice rats (*U* = 16.00, *p* < 0.05) (Figure 4E). However, no significant differences between the two groups in oleoylethanolamide (OEA) levels were detected (Figure 4D). These results demonstrate that male offspring from free-choice diet dams had lower levels of the main endocannabinoids, their direct metabolite, AA, as well as PEA in the hypothalamus as compared to controls at birth.

### Hippocampal endocannabinoid and N-acylethanolamide levels of male offspring at birth

Regarding hippocampal endocannabinoids and N-acylethanolamides (NAEs), U Mann Whitney test revealed a significantly decreased content of AEA (*U* = 5.00, *p* < 0.05) and PEA (*U* = 9.00. *p* < 0.05) in offspring from free-choice dams (Figures 4F,J, respectively). In contrast, there were no significant differences in the levels of 2-AG, AA and OEA between groups (Figures 4G–I respectively). These data indicate that male offspring from free choice dams had lower levels of anandamide and PEA.

### Effect of maternal diet on weight gain, caloric intake and body composition in male offspring

#### Effect of maternal diet on offspring weight gain in lactation period

**Figure 5:**
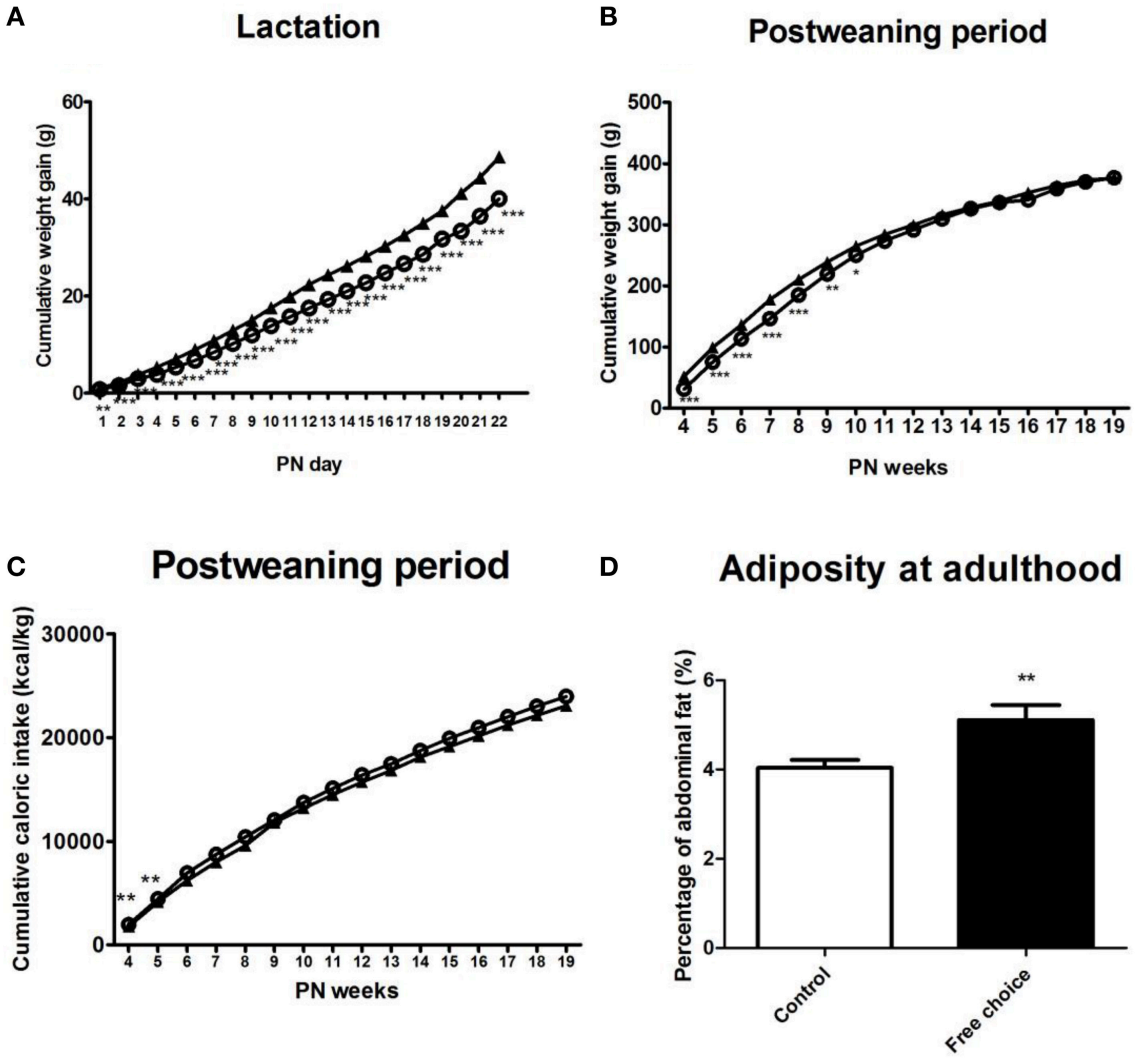
**Effects of maternal diet on male offspring development during lactation and post-weaning period**. Values are expressed as mean ± SEM. ^*^p < 0.05, ^**^p < 0.01, ^***^p < 0.001. Cumulative weight gain (g) of control offspring (solid triangles) and offspring from free-choice dams (open circles) during lactation and post-weaning period **(A,B)**. Cumulative caloric intake (kcal/kg) of control offspring (solid triangles) and offspring from free-choice dams (open circles) during post-weaning period **(C)**. Offspring adiposity at the 5th PN month **(D)**: control offspring (open bars) vs. offspring from free-choice dams (solid bars).

Repeated measures ANOVA indicated that offspring from free-choice dams continued being leaner than control offspring during the lactation period [*F*_(1, 71)_ = 44.914, *p* < 0.001] (data not shown). They also gained less weight during this period as revealed by repeated measures ANOVA [*F*_(1, 71)_ = 34.599, *p* < 0.001] (Figure 5A).

#### Effect of maternal diet on offspring weight gain and caloric intake in post-weaning period

After weaning (post-weaning period), repeated measures ANOVA showed a significant effect of the perinatal diet on male offspring weight as well as weight gain during the entire post-weaning period [*F*_(1, 28)_ = 4.55, *p* = 0.042, and *F*_(1, 30)_ = 4.277, *p* = 0.047, respectively]. Particularly, Bonferroni's analysis for comparisons among groups showed that offspring from free-choice dams gained significantly less weight than control offspring but only in the first 10 PN weeks, which is considered the pre-adult period [*F*_(1, 30)_ = 26.831, *p* < 0.001 in 4 PN week; *F*_(1, 30)_ = 26.519, *p* < 0.001 in 5 PN week; *F*_(1, 30)_ = 29.526, *p* < 0.001 in 6 PN week; *F*_(1, 30)_ = 36.472 in 7 PN week; *F*_(1, 30)_ = 20.652, *p* < 0.001 in 8 PN week; *F*_(1, 30)_ = 10.199, *p* < 0.01 in 9 PN week and *F*_(1, 30)_ = 4.723, *p* < 0.05 in 10 PN week] (Figure 5B).

Concerning the caloric intake, repeated measures ANOVA indicated that there was not a statistical difference between the two animal groups over the entire post-weaning period except in the 4th and 5th postnatal weeks, where offspring from free-choice dams showed higher caloric intake (kcal/kg) than controls [*F*_(1, 30)_ = 8.505, *p* = 0.007 and *F*_(1, 30)_ = 8.82, *p* = 0.006, respectively] (Figure 5C).

Taken together, these results demonstrate that male offspring from free-choice dams showed decreased body weight followed by a likely compensatory increase in caloric intake, which occurred only in the first phase of the post-weaning period. Indeed, in the adolescence period no difference between the two animal groups was detected.

#### Effect of maternal diet on offspring adiposity at adulthood

At the 5th PN month, male offspring from free-choice dams showed significantly higher abdominal fat than control offspring (*t* = 3.008, *p* < 0.01) (Figure 5D), although no significant difference in weight between groups was found.

### Effect of maternal diet on male offspring behavior

#### Anxiety-related responses: elevated plus maze test and open field test

**Figure 6:**
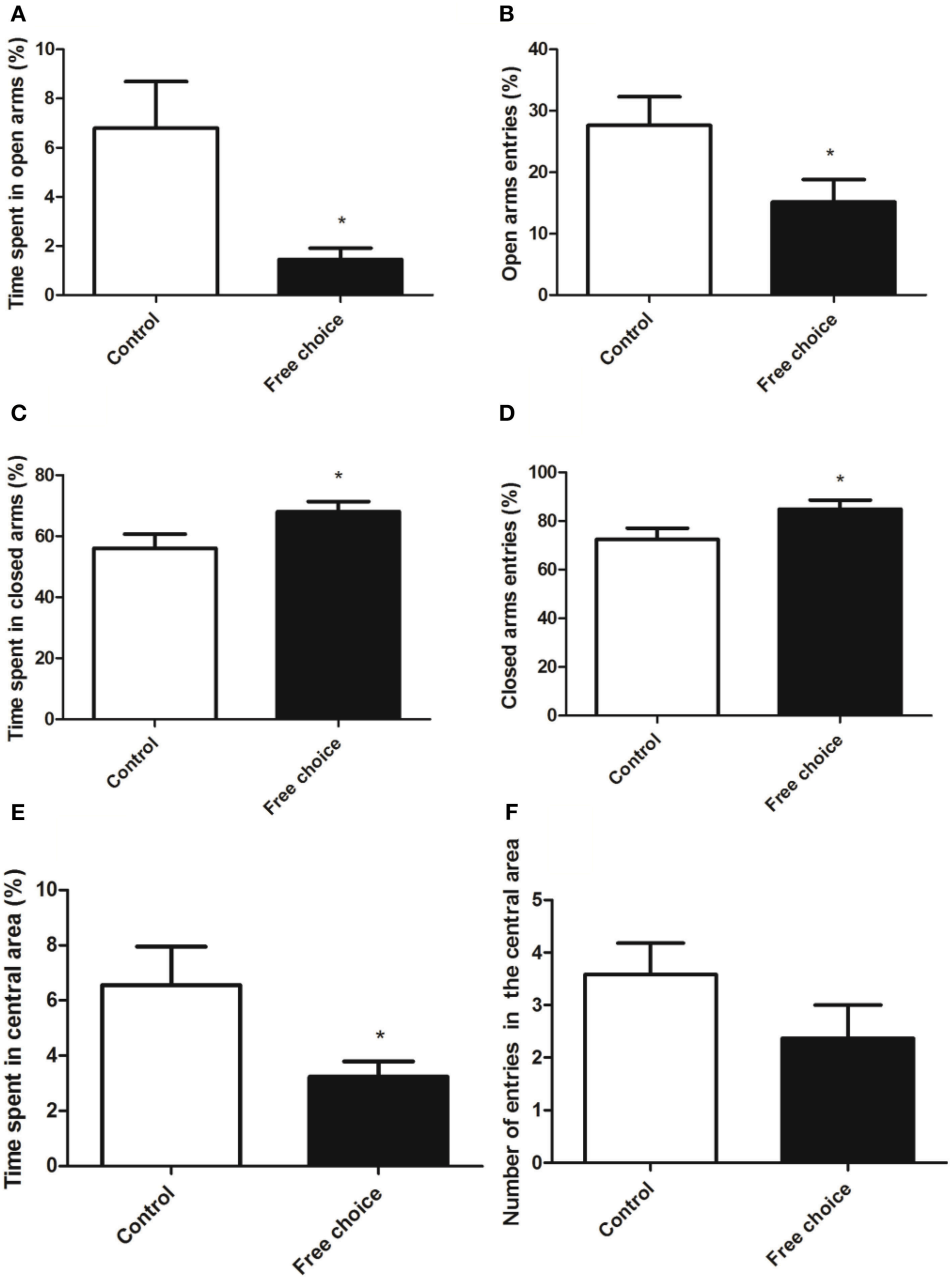
**Anxiety-related behavior in the elevated plus maze test and in the open field test of adolescent male animals born from mothers exposed to standard or highly palatable diet**. Values are expressed as mean ± SEM. ^*^p < 0.05. Open arm activity: percentage of time spent and percentage of entries in the open arms **(A,B)**. Closed arms activity: percentage of time and percentage of entries in the closed arms **(C,D)**. Data are expressed as percentage of total time or entries in the two respective compartments. **(E,F)** Percentage of time spent and number of entries into the center area of the open field.

Student's *t*-test and U Mann Whitney test analysis revealed significant differences in the anxiety-like behavior between the two animal groups. Indeed, offspring from free-choice dams spent significantly less time in the open arms of the elevated plus maze (*U* = 45.50, *p* < 0.05) and entered less often in the open arms as compared to control rats (*t* = 2.129, *p* < 0.05) (Figures 6A,B, respectively). Conversely, offspring from free-choice dams spent longer time in the closed arms of elevated plus maze (*t* = 2.153, *p* < 0.05) and also entered more times in the closed arms as compared to control rats (*t* = 2.129, *p* < 0.05) (Figures 6C,D, respectively).

Regarding the open field test, U Mann Whitney and Student's *t*-test analysis showed that offspring from free choice dams spent significantly less time in the central area of the arena (*U* = 31.00, *p* < 0.05), although no significant differences between groups were found in the number of entries in the center zone (*t* = 1.400, *p* = 0.17) (Figures 6E,F respectively).

These results indicate that male offspring from dams exposed to palatable food (free-choice diet) displayed higher anxiety-related behavior than control (standard diet) offspring.

#### Locomotor activity: open field test

Student's *t*-test analysis showed no significant differences between groups in the total distance traveled and mean speed in the open field test (Supplementary Figures 3A,B, respectively). These results suggest that perinatal diet does not affect locomotor activity in the offspring.

#### Chocolate preference test

**Figure 7:**
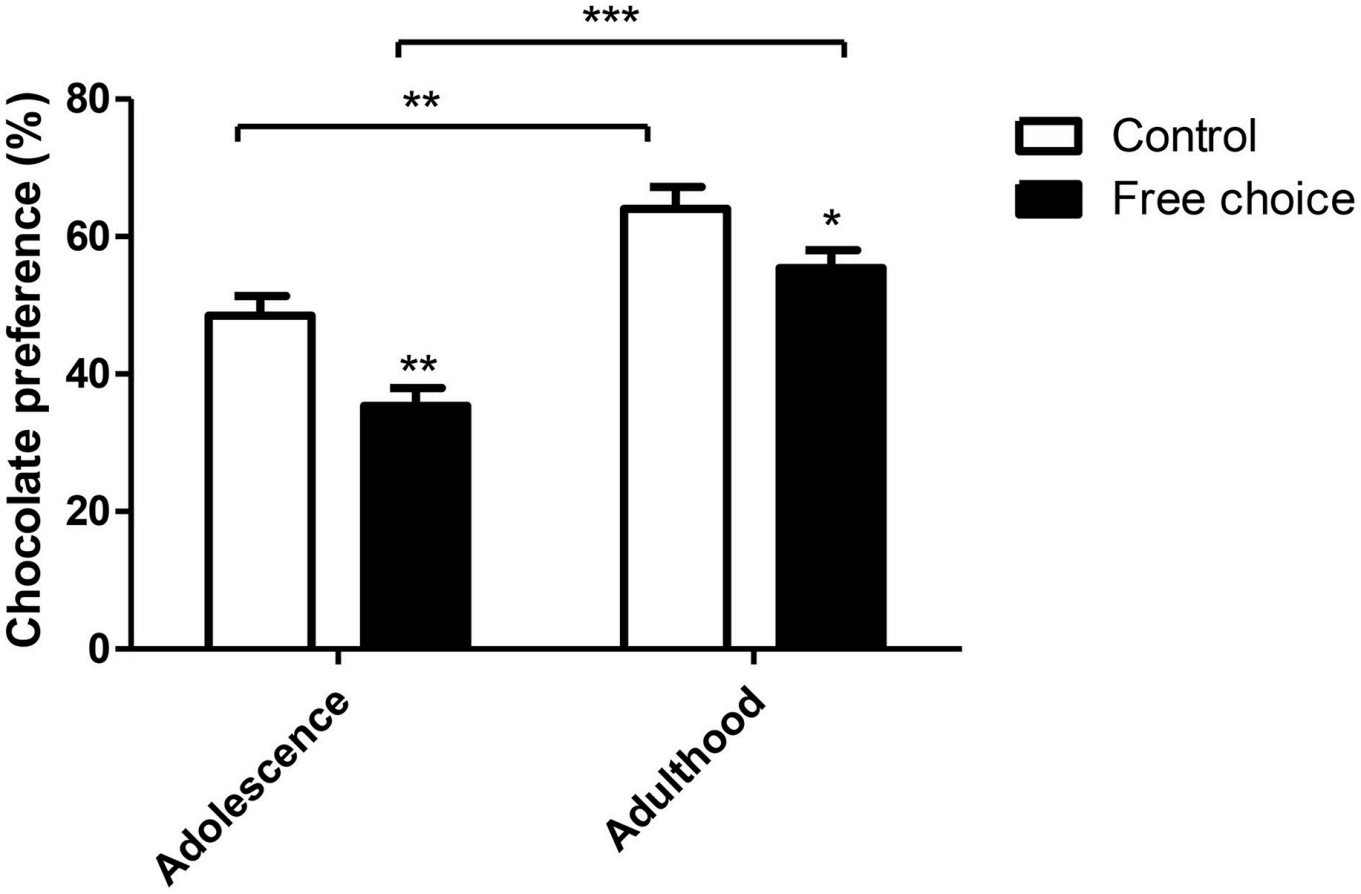
**Chocolate preference test in offspring**. Values are expressed as mean ± SEM. ^*^p < 0.05, ^**^p < 0.01, ^***^p < 0.001. Chocolate preference in adolescence and adulthood is expressed as percentage of chocolate eaten over total food intake.

Two-way ANOVA test performed on the chocolate preference test carried out in adolescence and adulthood, showed a statistically significant effect for both factors, animal group (type of perinatal diet) [*F*_(1, 59)_ = 14.848, *p* < 0.001] and age period (adolescence vs. adulthood) [*F*_(1, 59)_ = 39.557, *p* < 0.001]. However, no significant interaction between factors (diet type x age period) was found (Figure 7). Offspring from free-choice dams showed lower preference for chocolate food in both age periods as compared to controls. In addition, the difference between the two animal groups was higher in adolescence [*F*_(1, 29)_ = 11.591, *p* < 0.01] than in adulthood [*F*_(1, 31)_ = 4.455, *p* < 0.05]. Moreover, the preference for chocolate increased with the age in both groups as offspring from both control [*F*_(1, 27)_ = 12.910, *p* < 0.01] and free-choice [*F*_(1, 33)_ = 29.510, *p* < 0.001] dams had higher chocolate preference in adulthood than in adolescence (Figure 7). In conclusion, these results indicate that offspring from dams exposed to palatable food (free-choice diet) displayed less chocolate preference than control offspring.

## Discussion

The present study addressed the potential association of maternal consumption of a highly palatable food (free-choice), with (a) alterations in hypothalamic and hippocampal endocannabinoid levels in male offspring at birth, and (b) long-term phenotypic changes in adulthood. The results reported here clearly indicate that maternal over-nourishment with junk food is associated to long-term alterations in the offspring. The observed abnormalities in the pups included (a) low weight at birth, (b) altered contents of endocannabinoids and related acylethanolamides in both hypothalamus and hippocampus, and (c) long-term alterations in terms of abdominal adiposity and anxiety-like behaviors. As the endocannabinoid system has been implicated in the control of feeding, energy expenditure, adipose tissue physiology and the control of emotional behavior, we propose that the alterations in the hypothalamic/hippocampal endocannabinoid system can be linked to the phenotypic differences observed as a result of the type of maternal diet consumed.

### Maternal highly palatable diet alters hypothalamic/hippocampal endocannabinod levels in male offspring at birth

One of the main findings of the present study is the existence of alterations in the endocannabinoid contents in the brain of pups born from free-choice dams. As discussed below, these alterations might have potential implications in feeding (suckling behavior), adipogenesis, and might lead behavioral alterations such as anxiety. How maternal consumption of a cafeteria diet might affect brain endocannabinoids in the offspring? Arachidonic acid (20:4n-6), the precursor of the AEA and 2-AG, derives from dietary linoleic acid (18:2n-6), and it is reduced in the brain of the free-choice group offspring. It has been suggested that diets containing different polyunsaturated fatty acids, when given throughout adult life, could modify the levels of arachidonic acid and therefore, the content of endocannabinoids (Hansen and Artmann, 2008). The decrease in arachidonic acid in the offspring may have been determined by the decreased availability of linoleic acid in the maternal diet, and the reduced protein intake in the dams. Although we have not examined the specific composition of polyunsaturated fatty acids in the cafeteria diet (highly palatable diet), this diet contains a high percentage of fat, especially saturated fat (56.2% vs. 22.2%, Table 1). Several studies have shown changes in arachidonic acid levels or its derivatives depending on the saturated fatty acids composition of the diet. Indeed, a diet rich in saturated fat leads to decreased 2-AG and OEA levels when compared to a diet rich in oleic acid or arachidonic acid (Artmann et al., 2008). The presence of n-3 fatty acids could also impair arachidonic acid levels depending on the saturated fatty acid and linoleic acid content in the diet (Garg et al., 1990).

### Potential impact of reduced endocannabinoids in feeding behavior during lactation

Newborn pups from free-choice dams exhibited marked reductions of endocannabinoid levels in the hypothalamus and the hippocampus. It has been shown that the endocannabinoid system has a pivotal role in milk suckling, which might lead to alterations in growth during lactation, and consequently to low weight gain, as reported here (Figure 5). Decreased milk ingestion during the first days of life has been reported in cannabinoid CB_1_ receptor knockout mice (CB_1_-KO) and after pharmacological blockade of CB_1_ receptor in newborn pups (Fride et al., 2001, 2003). Thus, the activation of the CB_1_ receptor appears to be critical for the initiation of milk suckling. In line with this evidence, we noticed that litters from cafeteria diet-fed mothers showed less pronounced milkbands at birth (data not shown) and displayed lower levels of hypothalamic AEA, 2-AG and PEA than controls at PN day 0, which might have affected the suckling process. Therefore, we hypothesize that deficient suckling together with diminished milk supply and protein content could have altered weight gain in the pups during lactation. This hypothesis is supported by previous reports adopting a study design similar to ours (Bayol et al., 2007; Ong and Muhlhausler, 2011) showing that male pups from cafeteria diet-fed dams remained leaner even during lactation. Therefore, we can attribute the low weight gain to both an endocannabinoid-related decreased suckling and a delayed lactogenesis, as suggested for obese or overweight mothers (Rasmussen et al., 2001; Rasmussen and Kjolhede, 2004; Matias et al., 2014). Indeed, our study shows that dams exhibited higher abdominal adiposity at weaning. The combination of both factors could have led to a diminished milk intake by the pups and to a low protein intake, which consequently may have affected their early and late development.

Whether or not the hypothalamic endocannabinoid changes are a main factor in the low weight observed in free-choice offspring remains to be determined. Our data are in line with several previous works using pregnant rats exposed to junk food (cafeteria diet) (Bayol et al., 2007; Ong and Muhlhausler, 2011) or high fat diet (Guo and Jen, 1995; Langley-Evans, 1996; Howie et al., 2009). Both macrosomy and low weight at birth have been described in humans and rodents exposed to overnutrition (Baeten et al., 2001; Holemans et al., 2004; Boney et al., 2005; Bhattacharya et al., 2007; Samuelsson et al., 2008; Rajasingam et al., 2009). Moreover, these effects have been shown to depend on the fatty acid and saturated fat composition of the diet, and the duration of high-fat diet exposure (Hausman et al., 1991; Sullivan et al., 2011). Another important criterion to consider is the combination of high fat level consumption and low protein intake during pregnancy, as reported in several works (Bayol et al., 2007; Ong and Muhlhausler, 2011). Indeed, considering that the proteins present in the diet provide a supply of amino acids and essential nutrients, which are required for an adequate growth, animals exposed to a protein-restricted diet generate offspring with low body weight at birth (Bonatto et al., 2005; Feoli et al., 2006; Coupé et al., 2010). In our study, a low milk intake in the free-choice pups could have generated a state of protein malnutrition equivalent to that reported in these previous studies.

### Maternal highly palatable diet alters adiposity in adult offspring

The endocannabinoid system is involved in adipogenesis and fat accumulation (Matias and Di Marzo, 2007), however, to date, it has not been studied as a potential modulator of metabolic and behavioral programming. The reduced hypothalamic endocannabinoid levels found at birth may have had possible implications for the development of adiposity later in adulthood. However, considering that free-choice group offspring were fed with a standard diet after weaning, the decreased endocannabinoid levels at birth cannot be directly correlated to the development of adipogenesis in adulthood. Measurement of endocannabinoid levels in the offspring at different age periods in the future will provide important information to corroborate this notion. In any case, several works using dietary manipulations during perinatal exacerbates adiposity later in life (Bayol et al., 2008; Samuelsson et al., 2008; Shankar et al., 2008; Howie et al., 2009; Dahlhoff et al., 2014). Therefore, in our case, exposing animals to a standard chow diet at weaning may have raised the possibility to develop adiposity later in life. It is also interesting to note that offspring from cafeteria diet-fed dams weigh less in the neonatal period but reached the same weight as control dams at adolescence, as mentioned before. In line with this observation, an association between low birth weight and long term adiposity has been reported in both animal models and humans (Ravelli et al., 1976, 1999; Anguita et al., 1993; Valdez et al., 1994). Some factors implicated may be compensation after early malnutrition (Bieswal et al., 2006; Bol et al., 2009) and upregulation of the adipogenic signaling pathway, such as genes involved in adipocyte differentiation (Guan et al., 2005) and lipogenic transcription factors (Desai et al., 2008). These modifications can lead to adipocyte hypertrophy and increased lipid storage later in life. Whether this is an endocannabinoid-related response remains to be determined.

### Maternal highly palatable diet increases anxiety-related response in offspring: an endocannabinoid-related developmental effect?

Consistent with previous works (Sasaki et al., 2013), we found that motor activity was not impaired in offspring from highly palatable diet-fed mothers. However, maternal exposure to this diet results in clear anxiety-like behaviors in the offspring. Similar results have been reported in the offspring after maternal exposure to high-fat diet (Sullivan et al., 2010; Peleg-Raibstein et al., 2012; Sasaki et al., 2013), although several discrepancies have been reported in literature on this matter (Wright et al., 2011; Sasaki et al., 2014) probably due to methodological issues such as the intensity of the illumination used in the elevated plus maze test, or the composition of the diet given during the perinatal period. In this study we used a low protein cafeteria diet and maternal low protein diets have been associated to increased anxiety in offspring (Reyes-Castro et al., 2012).

The anxious phenotype observed in male offspring born from mothers exposed to the free-choice diet can be related to the alterations in brain endocannabinoids observed. Endocannabinoids are important modulators in the development of the fetal brain (Berrendero et al., 1999). Moreover, several studies have described important changes in endocannabinoid levels in the developmental brain as a result of dietary manipulations (Berger et al., 2001; Matias et al., 2003; D'Asti et al., 2010). As the endocannabinoid system plays a relevant role in processes such as cell fate, neuronal proliferation, migration, phenotype acquisition and synaptogenesis (Rodríguez de Fonseca et al., 1991; Berrendero et al., 1999; Keimpema et al., 2013), changes in brain endocannabinoid levels during development are likely capable to influence the final brain organization. Considering that, it has been suggested that alterations in endocannabinoid signaling by exposure to harmful stimuli such as THC during critical windows, could lead to alterations in neuronal wiring and consequently, to the risk of suffering behavioral or metabolic alterations later in life, depending on the localization and function of the circuit altered (Keimpema et al., 2013). The present study shows for first time that perinatal exposure to highly palatable diet leads to impairment in the main endocannabinoids and NAEs at birth in brain regions involved in the control of emotional behavior, such as hypothalamus and hippocampus (Navarro et al., 1997; Lutz, 2009). This is consistent with previous reports showing that dysregulation in the endocannabinoid system, due to long-term inadequate nutrition in early life stages, could lead to increased anxiety later in life (Lafourcade et al., 2011; Larrieu et al., 2012).

### Maternal highly palatable diet alters food preference in offspring

Finally, we addressed the question of whether the alterations found in the endocannabinoid system as a result of maternal diet might have an impact in food preferences in the offspring. It has been proposed that food preferences can be programmed during early life. The endogenous cannabinoid system has been implicated in food preference (Dore et al., 2014) and sweet taste perception (Niki et al., 2015) and dysregulation of this system during development may have an impact in food preferences. Indeed, it has been reported that maternal consumption of junk food increases the preference of the offspring for this type of food later in life (Bayol et al., 2007; Ong and Muhlhausler, 2011). In contrast, in our study, offspring from cafeteria diet-fed dams displayed lower chocolate preference compared to controls, although they were exposed to this type of meal through their mothers early in life. This discrepancy can be explained on the basis of a potential neophobic response, considering that in the chocolate preference test a novel palatable food was provided in a new environment. This neophobic response is in agreement with the anxious phenotype of these animals, and it has been previously reported, for instance, in offspring from dams exposed to high-fat diet (Peleg-Raibstein et al., 2012).

## Conclusions

We have demonstrated that maternal exposure to a highly palatable diet results in changes in newborn male offspring, such as low body weight and decreased levels of endocannabinoids and NAEs in hypothalamus and hippocampus. These changes are associated with long-lasting consequences, as increased adiposity, anxiety-like behavior and reduced food preferences in adulthood. This is the first study to propose diet-induced alterations in the endocannabinoid levels at birth as a potential mechanism associated to long-term phenotypic consequences. Because of the current epidemics of obesity in western societies, the present work might help to design human studies to investigate to which extent these findings can be translated to humans, considering that dietary supplement of essential fatty acids is currently used as a simple preventive intervention. Understanding why dietary manipulations modify hypothalamic and hippocampal endocannabinoid levels and whether these changes lead to permanent dysfunctions in the body still need to be further investigated to clarify the role of the endocannabinoid system in nutritional programming.

### Conflict of interest statement

The authors declare that the research was conducted in the absence of any commercial or financial relationships that could be construed as a potential conflict of interest.
