# Supplementary material for: Exposure to a Highly Caloric Palatable Diet During Pregestational and Gestational Periods Affects Hypothalamic and Hippocampal Endocannabinoid Levels at Birth and Induces Adiposity and Anxiety-Like Behaviors in Male Rat Offspring
Source: Front Behav Neurosci. 2016 Jan 6;9:339. doi: 10.3389/fnbeh.2015.00339 (PMC4701936; doi:10.3389/fnbeh.2015.00339)

Supplementary Figure 1. Nutritional intake of dams during pregnancy and lactation

Values are expressed as mean +/- SEM. *** p< 0.001. Cumulative intake (g/kg) of proteins, carbohydrates and fats in control (open bars) and free-choice (solid bars) dams at gestational day 20 gestation (A) and at lactation day 21 (B).

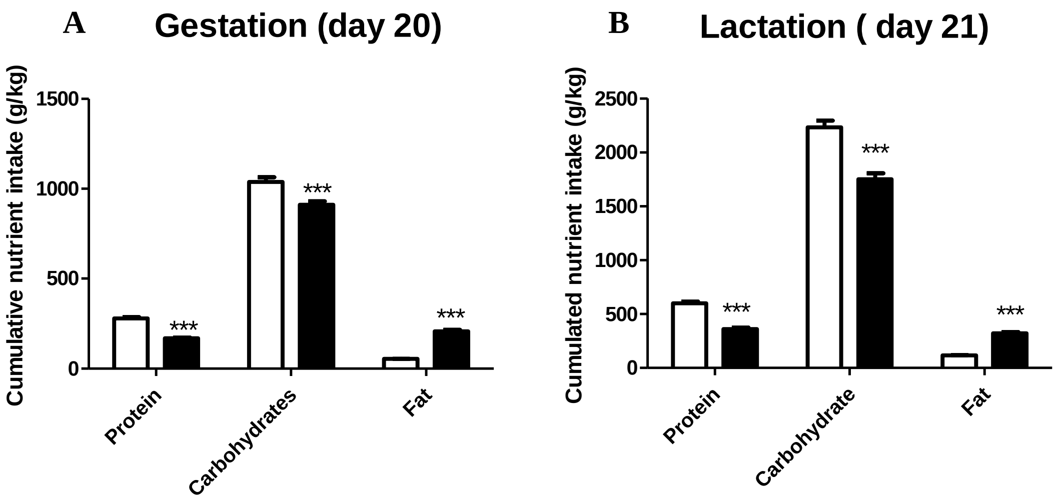

Supplementary Figure 2. Birth outcomes and mortality rate

Values are expressed as mean +/- SEM. ***p<0.001. Absolute body weight (g) (A) and litter size (B) at birth of offspring from control (open bars) and free-choice (solid bars) dams. Weight and size is shown for the total number of pups as wells as separetely for male and female pups (A, B). Survival curve during lactation (C).

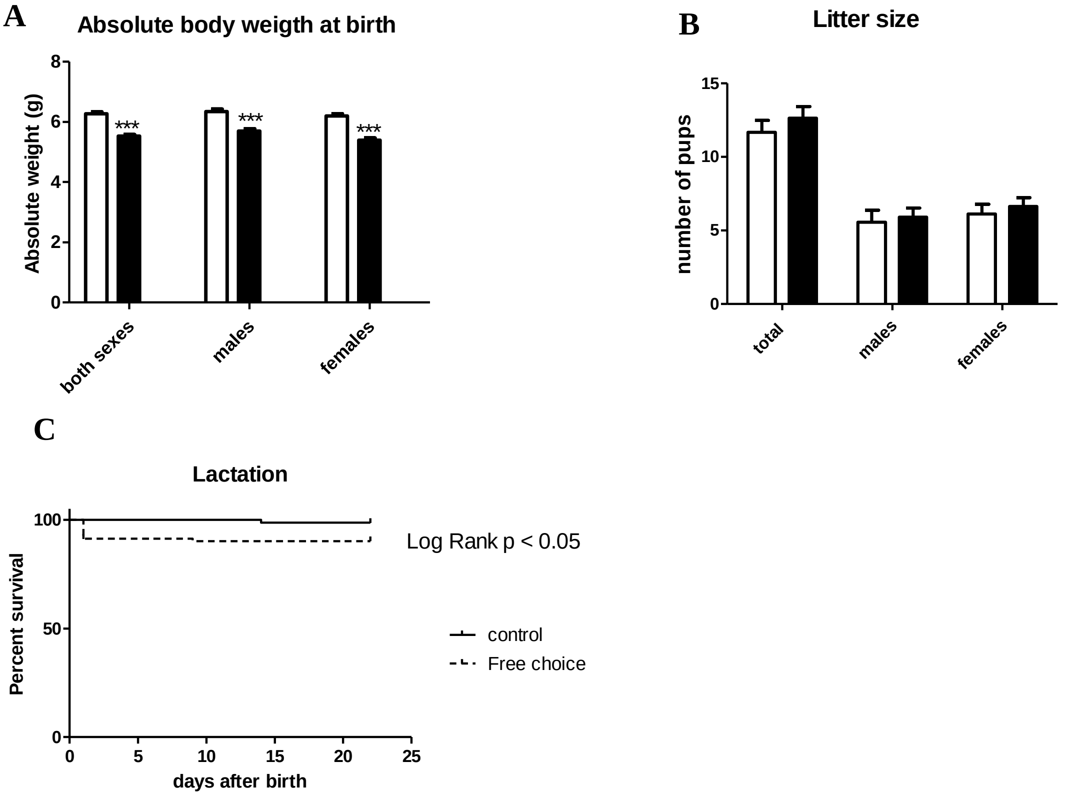

Supplementary Figure 3. Exploratory and locomotor activity in the open field test in adolescent male offspring

Values are expressed as mean +/- SEM. Total distance travelled (A) and mean speed (B) in offspring from control (open bars) and free-choice (solid bars) dams.

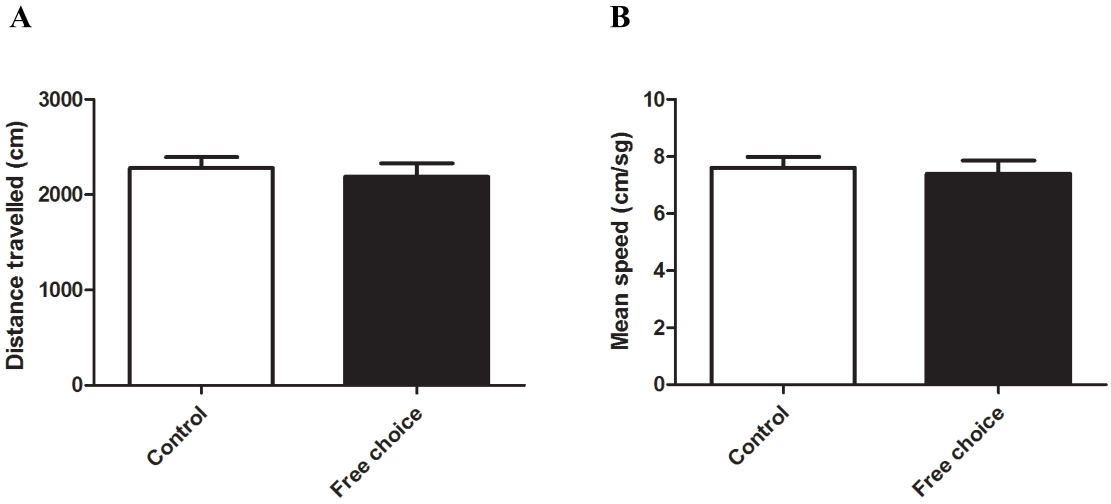

Supplement: Supplementary file 1 [file Presentation1.pdf]
